# The Alleviation of Photosynthetic Damage in Tomato under Drought and Cold Stress by High CO_2_ and Melatonin

**DOI:** 10.3390/ijms21155587

**Published:** 2020-08-04

**Authors:** Rong Zhou, Hongjian Wan, Fangling Jiang, Xiangnan Li, Xiaqing Yu, Eva Rosenqvist, Carl-Otto Ottosen

**Affiliations:** 1 Department of Food Science, Aarhus University, 8200 Aarhus N, Denmark; coo@food.au.dk; 2 Institute of Vegetables, Zhejiang Academy of Agricultural Sciences, Hangzhou 310021, China; hjwan@mail.zaas.ac.cn; 3 College of Horticulture, Nanjing Agricultural University, Nanjing 210095, China; jfl@njau.edu.cn (F.J.); xqyu@njau.edu.cn (X.Y.); 4 Northeast Institute of Geography and Agroecology, Chinese Academy of Sciences, Changchun 130102, China; lixiangnan@iga.ac.cn; 5 Department of Plant and Environmental Sciences, University of Copenhagen, 2630 Taastrup, Denmark; ero@plen.ku.dk

**Keywords:** tomato, elevated CO_2_ concentration, melatonin, drought stress, cold stress, recovery

## Abstract

The atmospheric CO_2_ concentration (a[CO_2_]) is increasing at an unprecedented pace. Exogenous melatonin plays positive roles in the response of plants to abiotic stresses, including drought and cold. The effect of elevated CO_2_ concentration (e[CO_2_]) accompanied by exogenous melatonin on plants under drought and cold stresses remains unknown. Here, tomato plants were grown under a[CO_2_] and e[CO_2_], with half of the plants pre-treated with melatonin. The plants were subsequently treated with drought stress followed by cold stress. The results showed that a decreased net photosynthetic rate (P_N_) was aggravated by a prolonged water deficit. The P_N_ was partially restored after recovery from drought but stayed low under a successive cold stress. Starch content was downregulated by drought but upregulated by cold. The e[CO_2_] enhanced P_N_ of the plants under non-stressed conditions, and moderate drought and recovery but not severe drought. Stomatal conductance (g_s_) and the transpiration rate (E) was less inhibited by drought under e[CO_2_] than under a[CO_2_]. Tomato grown under e[CO_2_] had better leaf cooling than under a[CO_2_] when subjected to drought. Moreover, melatonin enhanced P_N_ during recovery from drought and cold stress, and enhanced biomass accumulation in tomato under e[CO_2_]. The chlorophyll *a* content in plants treated with melatonin was higher than in non-treated plants under e[CO_2_] during cold stress. Our findings will improve the knowledge on plant responses to abiotic stresses in a future [CO_2_]-rich environment accompanied by exogenous melatonin.

## 1. Introduction

Climate change is expected to have a significant impact on agricultural production and food security [1,2]. Plants are subjected to various environmental stresses during their lifecycle, the frequency and intensity of which are increasing due to climate change [2]. For instance, drought and cold are significant factors that limit agricultural crop production [3,4]. Elevated CO_2_ concentration (e[CO_2_]) enhances plant photosynthesis and biomass even under adverse environmental conditions [5,6]. The e[CO_2_] can increase water-use efficiency and enhance plant growth, leading to higher water use [7]. On the one hand, Liu et al. (2019) reported that e[CO_2_] retarded tomato leaf’s response to progressive drought stress (DS) as reflected by the slow response of the net photosynthetic rate (P_N_) and stomatal conductance (g_s_) to DS under e[CO_2_] than control [CO_2_] [6]. On the other hand, plants grown under e[CO_2_] showed greater cold tolerance than control [CO_2_] [8]. Similarly, increasing [CO_2_] enhanced the photosynthetic electron transport system and ATP synthesis of wheat under cold stress (CS) [9]. The question is how tomatoes will react to successive abiotic stresses like drought and cold instead of individual stress in a future [CO_2_]-rich environment.

Melatonin, N-acetyl-5-methoxytryptamin, is a well-known hormone that plays alleviating roles in the plant response to abiotic stress, such as drought [10,11,12,13] and cold [14,15]. The addition of 0.1 mM melatonin through irrigation can alleviate oxidative stress due to DS and delay leaf senescence of apple [10]. Li et al. (2015) found that 0.1 mM melatonin through irrigation was involved in the regulation of reactive oxygen species (ROS) and abscisic acid (ABA) in *Malus* species exposed to drought [11]. Similarly, watering of 0.5 mM to wheat improved drought tolerance by enhancing the antioxidant capacity, alleviating membrane damage, as well as increasing gene transcription related to glutathione transferase and ascorbate [12]. Moreover, Posmyk et al. (2009) showed that 50 M melatonin application by hydropiming and osmoprining seeds protected the membrane structure of cucumber during chilling stress and recovery, which improved seed germination under a low temperature [14]. Spaying 1 mM melatonin benefited the plant growth of barley under a low temperature by enhancing the photosynthetic rate, processing a lower ROS burst, regulating carbohydrate metabolism, as well as upregulating the expression of key genes, such as rubisco [15,16]. Recently, Li et al. (2018) showed that melatonin enhanced carbon assimilation in wheat under e[CO_2_] and positive effects of melatonin in maternal wheat enhanced the cold tolerance of offspring seedlings [17]. However, the interacting effects of e[CO_2_] and exogenous melatonin on plants under successive abiotic stresses are unknown

Tomato is an important crop grown globally. The harvested area of tomato increased from 2.28 M ha in 1977 to 4.76 M ha in 2018 (http://www.fao.org). The individual effects of e[CO_2_] and melatonin on tomato were documented in previous studies. On the one hand, e[CO_2_] has been shown to have positive effects on tomato growth under abiotic stresses. For instance, e[CO_2_] together with decreased irrigation improve tomato water-use efficiency [18]. Liu et al. (2019) suggested that e[CO_2_] enhanced P_N_, increased the shoot C/N ratio, and retarded tomato responses to progressive soil drying [6]. On the other hand, melatonin was proved to be a crucial molecule involved in the tomato response to abiotic stresses [19,20,21]. For instance, the addition of 0.1 mM melatonin through both soaking seeds and irrigating tomato plants enhanced drought tolerance [19]. Spraying 0.1 mM melatonin induced thicker cutin and increased the cuticular wax level in tomato leaves that restricted water loss when plants were exposed to DS [20]. Furthermore, spraying 0.1 mM melatonin ameliorated cold damage and enhanced the ROS scavenging system of tomato plants [22]. Similarly, Yang et al. (2018) demonstrated that 0.1 mM melatonin both by leaf spray and root irrigation could alleviate cold (15 °C at daytime/6 °C nighttime for 3 days) damage on the tomato photosynthetic capacity [21]. In addition to positive effects on the vegetative part, 0.02 mM melatonin through irrigation eased heat-induced inhibition of tomato pollen [23].

**Figure 1:**
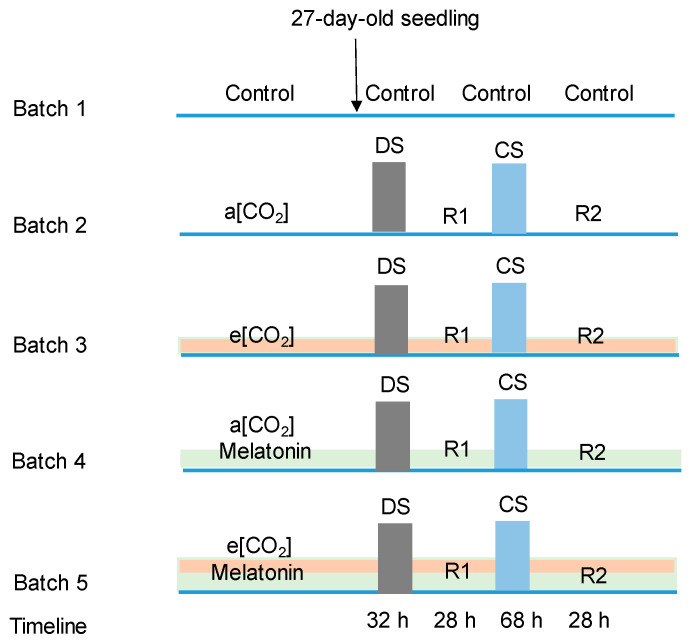
Treatment flow of the experiments. “a[CO_2_]” and “e[CO_2_]” indicate 400 and 800 ppm CO_2_ concentration, respectively. The batch marked with “melatonin” indicates the plants were treated by seven times of 1 mM melatonin. “DS”, drought stress, 25/20 °C + no irrigation; “CS”, cold stress, 12/12 °C + irrigation; “R1” and “R2”, recovery, 25/20 °C + irrigation.

Nevertheless, the interaction of e[CO_2_] and exogenous melatonin on tomatoes under successive drought and cold stress needs to be investigated in view of climate change. Tomatoes were subjected to DS followed by CS in association with e[CO_2_] and exogenous melatonin in this study (Figure 1), with the aim to clarify potential physiological regulation mediated by e[CO_2_] and melatonin. Our hypothesis was that (1) e[CO_2_] could have a positive influence to the response of tomato to successive stresses, and (2) exogenous application of melatonin might enhance the tolerance of tomato to drought and cold stress under e[CO_2_]. This study provides novel insights about the interactive effects of e[CO_2_] and exogenous melatonin on tomato physiology, which are needed for better prediction and regulation of crop growth under future e[CO_2_] climates.

## 2. Results

**Figure 2:**
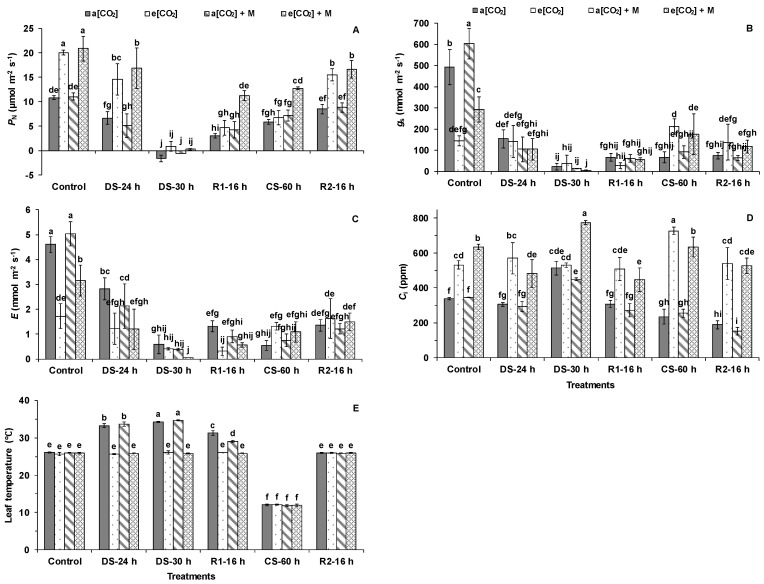
(**A**) Net photosynthetic rate (*P*_N_), (**B**) stomatal conductance (*g*_s_), (**C**) transpiration rate (E), (**D**) intracellular CO_2_ concentration (*C*_i_), and (**E**) leaf temperature in the first fully expanded leaves of tomato from the top during different treatments. “a[CO_2_]” and “a[CO_2_] + M” indicates 400 ppm CO_2_ concentration without and with melatonin application. “e[CO_2_]” and “e[CO_2_] + M” indicates 800 ppm CO_2_ concentration without and with melatonin application. “Control”, 25/20 °C + irrigation; “DS”, drought stress, 25/20 °C + no irrigation, “CS”, cold stress, 12/12 °C + irrigation; “R1” and “R2”, recovery, 25/20 °C + irrigation. The data represent average values ± SD (*n* = 3). The ANOVA was conducted within all the treatments at different stages. Different small letters showed significant differences (*p* < 0.05).

As shown in Figure 2A, P_N_ of plants under atmospheric CO_2_ concentration (a[CO_2_]) and a[CO_2_] + melatonin (M) was significantly lower than e[CO_2_] and e[CO_2_] + M during the control condition. This trend was maintained after 24 h of DS, even though DS decreased P_N_ of all four treatments. However, there was no difference in P_N_ of plants after 30 h of DS (Figure 2A). The P_N_ of plants after 16 h of R1 partially recovered, with the highest P_N_ of plants under e[CO_2_] + M (Figure 2A). Similarly, 60 h of CS decreased the P_N_ of plants as compared with the control, showing the highest P_N_ of plants under e[CO_2_] + M (Figure 2A). After 16 h of R2, P_N_ of plants under e[CO_2_] and e[CO_2_] + M was still significantly higher than under a[CO_2_] and a[CO_2_] + M (Figure 2A).

The e[CO_2_] and e[CO_2_] + M significantly decreased g_s_ and E (transpiration rate) of the plants compared with a[CO_2_] and a[CO_2_] + M during the control condition (Figure 2B,C). Compared with the controls, 24 h of DS, 30 h of DS, 16 h of R1, 60 h of CS, and 16 h of R2 significantly decreased g_s_ and E of the plants under the four treatments except for the plants under e[CO_2_] after 24 h of DS, 60 h of CS, and 16 h of R2 (Figure 2B,C). After 60 h of CS, g_s_ of the plants grown under e[CO_2_] and e[CO_2_] + M was significantly higher than a[CO_2_] and a[CO_2_] + M (Figure 2B). By comparison, E of the plants under a[CO_2_] and a[CO_2_] + M was significantly higher than that under e[CO_2_] and e[CO_2_] + M after 24 h of DS (Figure 2C). After 16 h of R1, E of the plants under a[CO_2_] was significantly higher than e[CO_2_] (Figure 2C). Generally, C_i_ (intracellular CO_2_ concentration) of the plants under e[CO_2_] and e[CO_2_] + M was higher than that under a[CO_2_] and a[CO_2_] + M during all the treatments except 30 h of DS (Figure 2D). The leaf temperature of the plants under a[CO_2_] and a[CO_2_] + M was higher than that under e[CO_2_] and e[CO_2_] + M after 24 h of DS, 30 h of DS, and 16 h of R1 (Figure 2E). The DS and R1 only significantly increased the leaf temperature of the plants under a[CO_2_] and a[CO_2_] + M (leaf temperature after 30 h of DS > after 24 h of DS > after 16 h of R1) (Figure 2E). The CS significantly decreased the leaf temperature of the plants under the four treatments, with no difference between them (Figure 2E).

**Figure 3:**
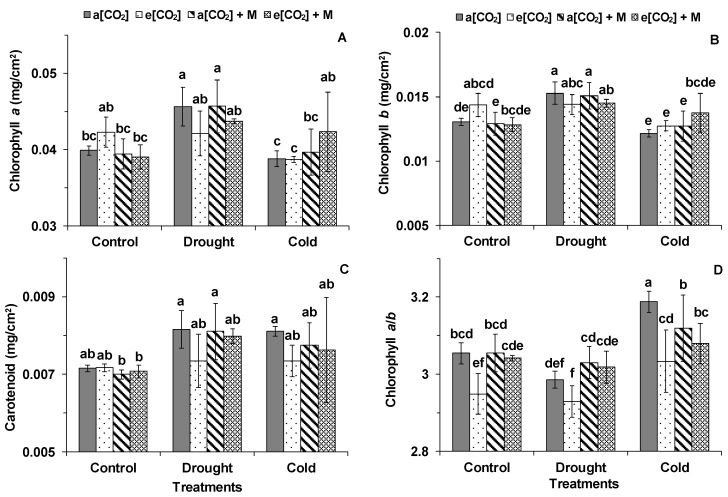
(**A**) Chlorophyll *a*, (**B**) chlorophyll *b*, (**C**) carotenoid, and (**D**) chlorophyll *a*/*b* in the first fully expanded leaves of tomato from the top during different treatments. “a[CO_2_]” and “a[CO_2_] + M” indicates 400 ppm CO_2_ concentration without and with melatonin application. “e[CO_2_]” and “e[CO_2_] + M” indicates 800 ppm CO_2_ concentration without and with melatonin application. “Control”, 25/20 °C + irrigation; “Drought”, 25/20 °C + no irrigation for 30 h; “Cold”, 12/12 °C + irrigation for 60 h. The data represent average values ± SD (*n* = 3). The ANOVA was conducted within all the treatments. Different small letters showed significant differences (*p* < 0.05).

The DS significantly increased the chlorophyll *a* and *b* content of the plants under a[CO_2_] and a[CO_2_] + M, while CS decreased the chlorophyll *a* and *b* content of the plants under e[CO_2_] compared with the controls (Figure 3A,B). The chlorophyll *a* content of the plants under e[CO_2_] + M was significantly higher than a[CO_2_] and a[CO_2_] + M after CS (Figure 3A). The DS induced a higher carotenoid content under a[CO_2_] + M as compared with the control (Figure 3C). After CS, chlorophyll *a*/*b* of the plants under a[CO_2_] and a[CO_2_] + M was significantly higher than their respective controls (Figure 3D). Chlorophyll *a*/*b* of the plants under a[CO_2_] and a[CO_2_] + M was significantly higher than under e[CO_2_] during the control and CS, while that of the plants under a[CO_2_] + M and e[CO_2_] + M was higher than under e[CO_2_] during DS (Figure 3D).

**Figure 4:**
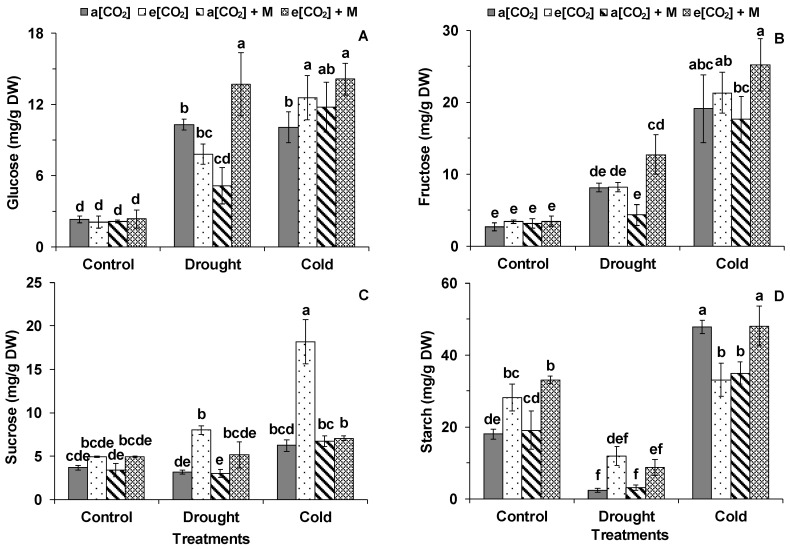
(**A**) Glucose, (**B**) fructose, (**C**) sucrose, and (**D**) starch in the first fully expanded leaves of tomato from the top during different treatments. Treatments are the same as in Figure 3. The data represent average values ± SE (*n* = 3). The ANOVA was conducted within all the treatments. Different small letters showed significant differences (*p* < 0.05).

The DS and CS significantly increased the glucose content for all treatments except a[CO_2_] + M during DS (Figure 4A). The glucose content of the plants under e[CO_2_] + M was significantly higher than the other three treatments after DS, while that of the plants under e[CO_2_] and e[CO_2_] + M was higher than under a[CO_2_] after CS (Figure 4A). The DS only significantly increased the fructose content of the plants under e[CO_2_] + M, while CS increased the fructose content of the plants under the four treatments in comparison with the respective controls (Figure 4B). After both DS and CS, the fructose content of the plants under e[CO_2_] + M was significantly higher than that under a[CO_2_] + M (Figure 4B). The sucrose content of the plants under e[CO_2_] was significantly higher than that under a[CO_2_] and a[CO_2_] + M after DS (Figure 4C). By comparison, the sucrose content of the plants under e[CO_2_] significantly increased as compared with the other three treatments after CS (Figure 4C). The DS significantly decreased the starch content, while CS increased the starch content as compared with the respective controls except for the plants under e[CO_2_] after CS (Figure 4D). The starch content under e[CO_2_] and e[CO_2_] + M was significantly higher than that under a[CO_2_] for the controlled plant, while that of the plants under a[CO_2_] and e[CO_2_] + M was higher than the other two treatments after CS (Figure 4D). 

The F_v_/F_m_ (maximum quantum efficiency of photo system II or PSII) of the plants under e[CO_2_] was lower than that under the other three treatments after 30 h of DS and 60 h of CS (Supplementary Material Figure S1). The F_q_’/F_m_’ (quantum yield of PSII) and ETR (electron transport rate) of plants under e[CO_2_] after R1 significantly decreased compared with controls, which was lower than that under a[CO_2_] after CS (Supplementary Material Figure S2A,B). The R1 decreased q_L_ (fraction of open PSII centers) under all treatments, but increased NPQ (non-photochemical quenching) of the plants under e[CO_2_] (Supplementary Material Figure S2C,D). The q_L_ of the plants under a[CO_2_] + M was higher than that under e[CO_2_], while NPQ of the plants under e[CO_2_] was higher than the other three treatments after R1 (Supplementary Material Figure S2C,D). Moreover, CS showed lower q_L_ under e[CO_2_] + M but a higher NPQ of the plants under the four treatments (Supplementary Material Figure S2C,D).

**Figure 5:**
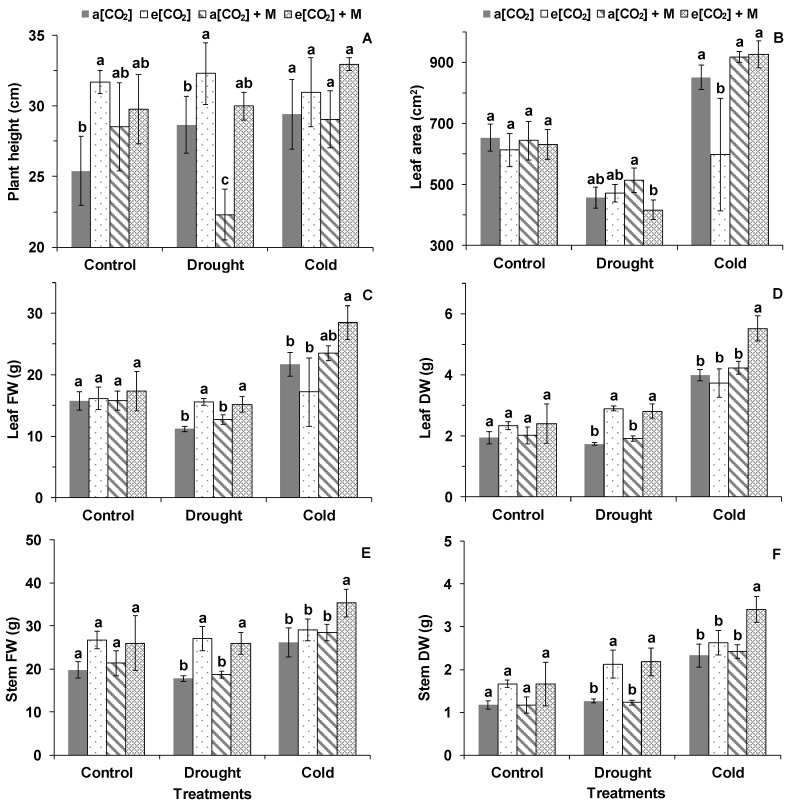
(**A**) Plant height, (**B**) leaf area, (**C**) leaf FW and (**D**) leaf DW, (**E**) stem FW, and (**F**) stem DW of tomato during the treatments. FW and DW are the abbreviations of fresh and dry weight, respectively. Treatments are the same as in Figure 3. The data represent average values ± SD (*n* = 3). Different small letters showed significant differences (*p* < 0.05).

**Figure 6:**
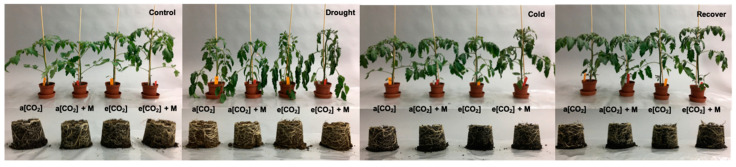
Plant morphology of tomato under different treatments. “a[CO_2_]” and “a[CO_2_] + M” indicates 400 ppm CO_2_ concentration without and with melatonin application. “e[CO_2_]” and “e[CO_2_] + M” indicates 800 ppm CO_2_ concentration without and with melatonin application. “Control”, 25/20 °C + irrigation; “Drought”, 25/20 °C + no irrigation for 32 h; “Cold”, 12/12 °C + irrigation for 68 h; “Recover”, 25/20 °C + irrigation for 24 h after cold stress.

Plant height increased under e[CO_2_] compared with a[CO_2_] under the control condition (Figure 5A). The leaf area of the plants under a[CO_2_] + M was bigger than that under e[CO_2_] + M after DS, while the leaf area under e[CO_2_] was smaller than the other three treatments after CS (Figure 5B). The FW (fresh weight) and DW (dry weight) of leaf and stem under a[CO_2_] and a[CO_2_] + M was lower than that under e[CO_2_] and e[CO_2_] + M after DS (Figure 5C−F). Generally, FW and DW of the leaf and stem under e[CO_2_] + M was the highest among the four treatments after CS (Figure 5C−F). Plant response regarding the morphology after DS was identical in all treatments with wilted leaves; however, there were no macroscopic symptoms of the plants after DS and R2 (Figure 6).

## 3. Discussion

Due to the increase in the frequency and severity of abiotic stresses [2] in field crops and increased a[CO_2_], it is urgent to understand how plants respond to complex environmental changes and potential alleviation methods. The effect of melatonin on animals has been widely studied compared with plants and the understanding of melatonin’s role in plants is just starting to emerge [24]. Previous studies primarily focused on the effect of melatonin on plants under a[CO_2_] and abiotic stresses [10,12,13]. The question is how melatonin affects plants under abiotic stresses accompanied by e[CO_2_].

### 3.1. Effects of e[CO_2_] on Tomato Photosynthesis

**Figure 7:**
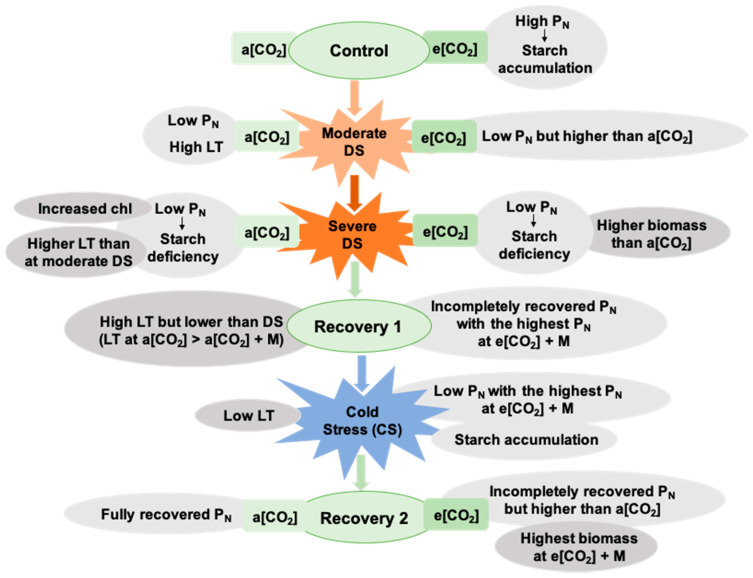
Schematic diagram of the main findings.

The decreased P_N_ of tomato was aggravated with a prolonged water deficit, which partially recovered when the irrigation restarted and remained low under CS (Figure 7). On the one hand, in accordance with previous studies [25,26,27,28], e[CO_2_] enhanced the P_N_ and starch accumulation under non-stressed conditions (Figure 7). On the other hand, e[CO_2_] can ease the damage caused by abiotic stresses, such as DS [29,30]. The e[CO_2_] reduced the effect of drought on grasses and legumes by decreasing H_2_O_2_ production and increasing molecular antioxidants [29]. Furthermore, e[CO_2_] mitigated the effect of reduced irrigation on tomato fruit yield [30]. We found that the alleviating effect of e[CO_2_] on the P_N_ only occurred in tomato under a moderate water deficit but not under severe DS (Figure 7). 

In accordance with previous studies, e[CO_2_] decreased g_s_ of the tomatoes under the control condition [31,32]. The trends of E positively corresponded to g_s_ under the control, with lower E under e[CO_2_]. However, this trend disappeared under the DS and recovery stages. Drought and cold stress reduced g_s_ and E but to a lesser degree under e[CO_2_] than a[CO_2_], resulting in a lower leaf temperature and better leaf cooling during DS under e[CO_2_] than a[CO_2_] (Figure 7). This indicated the leaf temperature was well-controlled through stomatal regulation in the plants grown under e[CO_2_] even when there were adverse environmental factors, such as a moderate and severe water deficit. These could partially explain why e[CO_2_] has a mitigation effect on tomato photosynthesis under DS. 

### 3.2. The Melatonin Played Positive Roles in Tomato Plants Grown Under Cold and e[CO_2_]

A positive effect of melatonin application was seen in tomato when irrigation restarted, with better leaf cooling under a[CO_2_] + M than a[CO_2_] (Figure 7). By comparison, P_N_ partially recovered, with the highest P_N_ under e[CO_2_] + M, when irrigation restarted and this trend was kept during the CS period, resulting in the highest plant biomass under e[CO_2_] + M after recovery (Figure 7). Previous studies have shown that melatonin application can enhance P_N_ of plants, including tomato, under CS and a[CO_2_] by reducing the damage of the low-temperature conditions on the photosynthetic apparatus and protecting the thylakoid membrane [21,22]. Our study provided proof that melatonin played a positive role in photosythesis protection during DS recovery and CS, and thereby benefits biomass accumulation in tomato under e[CO_2_]. 

Melatonin played roles in the regulation of chlorophyll loss and synthesis for the plants grown under abiotic stresses [33,34]. Li et al. (2018) found that exogenous application of melatonin can increase the chlorophyll content and delay the leaf senescence of wheat (Chl b-deficient mutant *ANK32B*) under e[CO_2_] [17]. In accordance, the chlorophyll *a* content of the tomatoes treated by melatonin was higher than non-treated plants under e[CO_2_] during CS. 

The effects of melatonin on downregulating chlorophyll breakdown or accelerating its synthesis during abiotic stresses were clear under e[CO_2_]. Moreover, chlorophyll *a*/*b* in the plants treated by melatonin was higher under e[CO_2_] during DS but lower under a[CO_2_] during CS compared with controls without melatonin. Li et al. (2018) [17] showed that alteration in chlorophyll *a*/*b* induced by melatonin was not seen during non-stressed conditions. Chlorophyll *a*/*b* could indicate the degree of leaf damage caused by oxidative stress [35]. An increase in chlorophyll *a*/*b* of tomato plants under e[CO_2_] + M compared with e[CO_2_] during the water deficit showed the alleviating effects of melatonin on the oxidative damage of tomato plants. Li et al. (2015) suggested that melatonin pre-treatment enhanced the drought tolerance of *Malus* species under a[CO_2_] through downregulation of ABA, better leaf water conservation, stable chlorophyll content, and increased P_N_^11^. Liu et al. (2015) concluded that melatonin pretreatment could improve tomato drought tolerance by increasing the photochemical efficiency and protecting against oxidative damage [19]. Furthermore, Ding et al. (2018) found that melatonin pretreatment could induce thick cutin, increase the cuticular wax level, and enhance wax gene expression in tomato leaves under DS, which explains how melatonin improves tomato drought tolerance through the limitation of leaf water loss [20]. Shi et al. (2015) demonstrated the positive role of melatonin treatment in bermudagrass responding to abiotic stresses, including drought, cold, and salt, through physiological, metabolomics, and transcriptomic regulation [36]. However, the response of tomato at the reproductive stage to abiotic stresses and melatonin needs further study in order to check how plant production was affected. More importantly, the underlying mechanism, especially molecular pathways concerning how melatonin works in plants, need to be further investigated.

## 4. Materials and Methods

### Growth Environmental Condition and Treatments

Seeds of tomato cultivar “Qianxi” (Known-you seed co. LTD, Taiwai, China) were sown in plastic pots with a 9-cm height and 11-cm diameter. This cultivar was chosen since it has been popular in the Chinese market for more than 10 years, which has good market prospects. Pots were filled by Pindstrup 2 (Pindstrup Mosebrug A/S, Ryomgaard, Denmark). Relative humidity was 43–55% and 49–63% and air temperature was 23 and 16 °C for day and night, respectively, in two rooms of a greenhouse. Light intensity was 150–300 μmol m^−2^ s^−1^ photosynthetic photon flux density (PPFD) with artificial light (FL300 SUNLIGHT, Søndersø, Denmark and SON-T Philips MASTER Green Power with 600W, Amsterdam, Netherlands) on when the light intensity was below 150 μmol m^−2^ s^−1^ PPFD. The difference in the environmental conditions between the two rooms was the CO_2_ concentration. Half plants grew in room 1 under the 400 ppm CO_2_ concentration (a[CO_2_]) and the other half of the plants grew in room 2 under the 800 ppm CO_2_ concentration (e[CO_2_]). On day 19 after sowing, nutrition solution (pH = 6, EC = 2.18, K = 275 ppm, N = 191 ppm, P = 35 ppm) was applied to the plants every day. On day 19, 22, and 25 after sowing, half of the seedling were sprayed (10 mL per plant) and irrigated (15 mL per plant) by 1 mM melatonin in the afternoon at 16:00 (a[CO_2_] + M and e[CO_2_] + M). The 25-day-old seedlings in room 1 were transferred to chamber 1 with a[CO_2_] and those in room 2 were transferred to chamber 2 with e[CO_2_]. The environmental parameters of two chambers were set to 25/20 °C (15 h day/9 h night), 60% relative humidity, and 300 μmol m^−2^ s^−1^ PPFD during daytime. The parameter settings of the two chambers were the same except the CO_2_ concentration. Each chamber had 36 seedlings and half of the seedlings were sprayed by melatonin. All seedlings were irrigated by the same nutrition solution twice a day by a flooding bench for 10 min at 8:00 and 16:00. Half of the 26-day-old plants were continuously sprayed (15 mL per plant) and irrigated (50 mL per plant) by 1 mM melatonin for the fourth time at 16:00 before stress. Then, the 27-day-old plants were subjected to DS by withdrawing irrigation from 8:00 for 32 h. The 28-day-old plants were irrigated at 16:00 as the recover stage 1 (R1) from DS for 28 h. Half of the 28-day-old and 29-day-old plants were continuously sprayed (15 mL per plant) and irrigated (100 mL per plant) by 1 mM melatonin at 16:00. Afterwards, the 29-day-old plants were subjected to CS (12/12 °C, day/night) from 20:00 for 68 h. The 32-day-old plants were subjected to a normal temperature (25/20 °C, day/night) from 16:00 as the recover stage 2 (R2) from CS. Half of the 32-day-old plants were continuously sprayed (15 mL per plant) and irrigated (100 mL per plant) by 1 mM melatonin at 16:00 during R2. In total, melatonin was applied for seven times. Control plants were sprayed and irrigated by the same amount of ddH_2_O (double-distilled water) as melatonin solution throughout the experiment. The first fully expanded leaf from the top was chosen for measurements.

Photosynthesis parameters: P_N_, g_s_, E, C_i_, and leaf temperature were measured using a portable photosynthesis system (CIRAS-2, PP Systems, Amesbury, USA). Measurements with three replicates were taken for the plants under control (before stress), DS for 24 h and 30 h, R1 for 16 h, CS for 60 h, and R2 for 16 h. We started to take records until five parameters were stable. The mean of the last six values were averaged and considered as the final results.

Measurements of the chlorophyll and carbohydrate content were taken from the plants under the control before stress, under DS for 30 h, and under CS for 60 h with three replicates. Leaf disks were punched using a cork borer and then the samples were immersed in 95% 4 °C ethanol for chlorophyll content measurements. Meanwhile, the samples were freeze-dried, ground, and weighed for carbohydrate content measurements [37].

Leaves was dark-adapted using a leaf clip for 25 min. Leaf F_v_/F_m_ was detected using Handy PEA (Hansatech Instrument, King’s Lynn, England). Measurements with four replicates were taken under the control (before stress), DS for 30 h, R1 for 16 h, CS for 60 h, and R2 for 16 h.

Plants were dark-adapted in a dark room for 20 min before quenching analysis. The F_q_’/F_m_’, q_L_, NPQ, and ETR of the plants were detected using MINI-PAM (Walz, Effeltrich, Germany) operated with WinControl 3 software (Walz, Effeltrich, Germany). A PPFD of 300 μmol m^−2^ s^−1^ during measurements was provided with an external light source (Schott KL 1500, Göttingen, Germany) through fiber optics. Measurements with three replicates were taken under the control (before stress), R1 for 16 h, CS for 60 h, and R2 for 16 h.

Plant growth parameters with three replicates were measured when the plants were grown under the control before stress, DS for 32 h, and CS for 68 h. Plant height from the cotyledonary node to growth point was recorded using a ruler. Leaf area was detected using a leaf area meter (3100, LI-COR, Lincoln, Nebraska, USA). Finally, FW of the leaf and stem was measured. After 48 h of drying at 80 °C, DW of the leaf and stem was measured.

The data were analyzed using analysis of variance (ANOVA) by SPSS 16.0 (SPSS Inc. Chicago, IL, USA).

## Supplementary Materials

Supplementary materials can be found at https://www.mdpi.com/1422-0067/21/15/5587/s1. Figure S1: The Fv/Fm in the first fully expanded leaves of tomato during different treatments. “a[CO_2_]” and “a[CO_2_] + M” indicates 400 ppm CO_2_ concentration without and with melatonin application. “e[CO_2_]” and “e[CO_2_] + M” indicates 800 ppm CO_2_ concentration without and with melatonin application. “Control”, 25/20 °C + irrigation; “DS-30 h”, 25/20 °C + no irrigation for 30 h; “CS-60 h”, 12/12 °C + irrigation for 60 h; “R1-16 h” and “R2-16 h”, 25/20 °C + irrigation for 16 h. The data represent average values ± SD (*n* = 4). Different small letters showed significant differences (*p* < 0.05); Figure S2: Chlorophyll fluorescence measured under a PPFD of 300 μmol m^−2^ s^−1^ at room temperature in tomato plants during different treatments. Different sub-graphs indicated (A) quantum efficiency of PSII (Fq’/Fm’), (B) electron transport rate (ETR), (C) fraction of open PSII centers (qL) and (D) non-photochemical quenching (NPQ). “a[CO_2_]” and “a[CO_2_] + M” indicates 400 ppm CO_2_ concentration without and with melatonin application. “e[CO_2_]” and “e[CO_2_] + M” indicates 800 ppm CO_2_ concentration without and with melatonin application. “Control”, 25/20 °C + irrigation; “R1-16 h”, 25/20 °C + irrigation for 16 h after 30 h of drought stress; “CS”, 12/12 °C + irrigation; “R2-16 h”, 25/20 °C + irrigation for 16 h after CS. The data represent average values ± SD (*n* = 3). Different small letters showed significant differences (*p* < 0.05).
