# Supplementary material for: The Alleviation of Photosynthetic Damage in Tomato under Drought and Cold Stress by High CO2 and Melatonin
Source: Int J Mol Sci. 2020 Aug 4;21(15):5587. doi: 10.3390/ijms21155587 (PMC7432885; doi:10.3390/ijms21155587)
Supplement: Supplementary file 1 [file ijms-21-05587-s001.pdf]

Supplementary Materials:

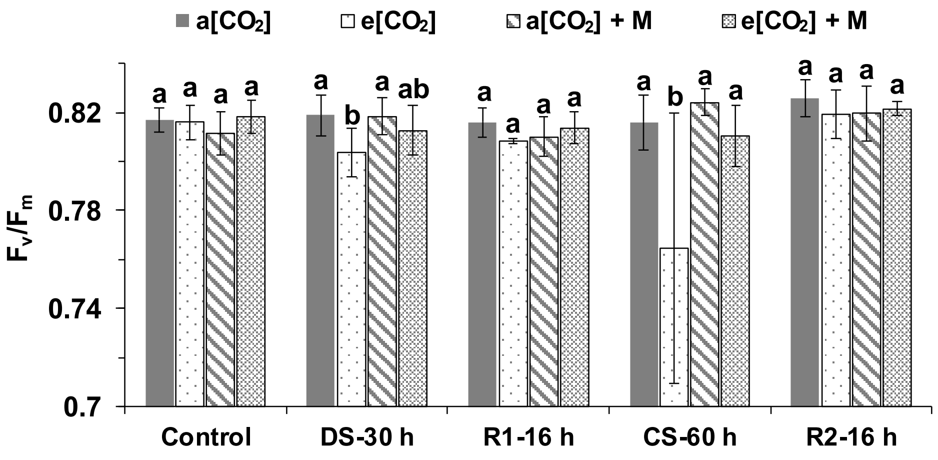

Supplementary Figure S1. The F_v/F_m in the first fully expanded leaves of tomato during different treatments. "a[CO_2]" and "a[CO_2] + M" indicates 400 ppm CO_2 concentration without and with melatonin application. "e[CO_2]" and "e[CO_2] + M" indicates 800 ppm CO_2 concentration without and with melatonin application. "Control", 25/20 °C + irrigation; "DS-30 h", 25/20 °C + no irrigation for 30 h; "CS-60 h", 12/12 °C + irrigation for 60 h; "R1-16 h" and "R2-16 h", 25/20 °C + irrigation for 16 h. The data represent average values ± SD (n = 4). Different small letters showed significant differences (p < 0.05).

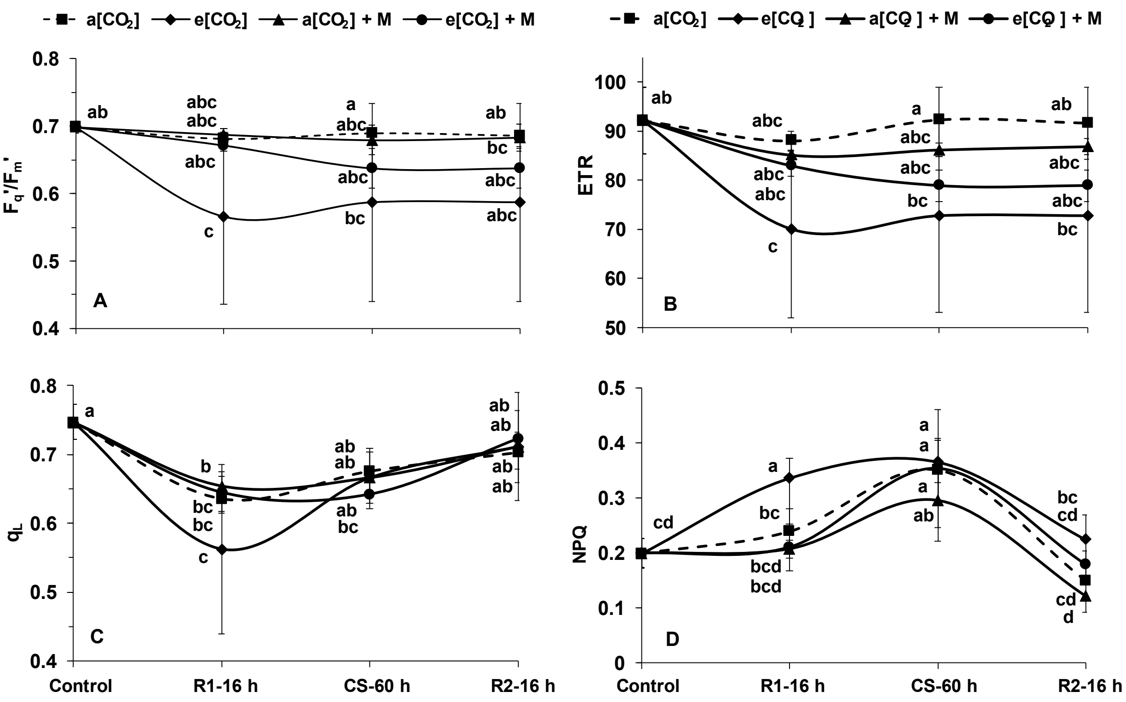

Supplementary Figure S2. Chlorophyll fluorescence measured under a PPFD of 300 μmol m^{-2} s^{-1} at room temperature in tomato plants during different treatments. Different sub-graphs indicated (**A**) quantum efficiency of PSII (F_q'/F_m'), (**B**) electron transport rate (ETR), (**C**) fraction of open PSII centers (q_L) and (**D**) non-photochemical quenching (NPQ). "a[CO_2]" and "a[CO_2] + M" indicates 400 ppm CO_2 concentration without and with melatonin application. "e[CO_2]" and "e[CO_2] + M" indicates 800 ppm CO_2 concentration without and with melatonin application. "Control", 25/20 °C + irrigation; "R1-16 h", 25/20 °C + irrigation for 16 h after 30 h of drought stress; "CS", 12/12 °C + irrigation; "R2-16 h", 25/20 °C + irrigation for 16 h after CS. The data represent average values ± SD (n = 3). Different small letters showed significant differences ($p < 0.05$).
